# Recent Progress in Enhancing Poly(Lactic Acid) Stereocomplex Formation for Material Property Improvement

**DOI:** 10.3389/fchem.2020.00688

**Published:** 2020-08-20

**Authors:** Fuhong Luo, Alexander Fortenberry, Jie Ren, Zhe Qiang

**Affiliations:** ^1^Department of Polymeric Materials, School of Materials Science and Engineering, Institute of Nano and Biopolymeric Materials, Tongji University, Shanghai, China; ^2^School of Polymer Science and Engineering, The University of Southern Mississippi, Hattiesburg, MS, United States

**Keywords:** sustainable, biodegradability, structure-property relationship, crystallization, advanced manufacturing

## Abstract

The production and utilization of polymers have been widely implemented into diverse applications that benefit modern human society, but one of the most valuable properties of polymers, durability, has posed a long-standing environmental challenge from its inception since plastic waste can lead to significant contamination and remains in landfills and oceans for at least hundreds of years. Poly(lactic acid) (PLA) derived from renewable resources provides a sustainable alternative to traditional polymers due to its advantages of comparable mechanical properties with common plastics and biodegradability. However, the poor thermal and hydrolytic stability of PLA-based materials limit their potential for durable applications. Stereocomplex crystallization of enantiomeric poly (l-lactide) (PLLA) and poly (d-lactide) (PDLA) provides a robust approach to significantly enhance material properties such as stability and biocompatibility through strong intermolecular interactions between L-lactyl and D-lactyl units, which has been the key strategy to further PLA applications. This review focuses on discussing recent progress in the development of processing strategies for enhancing the formation of stereocomplexes within PLA materials, including thermal processing, additive manufacturing, and solution casting. The mechanism for enhancing SC formation and resulting material property improvement enabled by each method are also discussed. Finally, we also provide the perspectives on current challenges and opportunities for improving the understanding of processing-structure-property relationship in PLA materials that could be beneficial to their wide practical applications for a sustainable society.

## Introduction

Traditional petroleum-derived plastics have been “ideal” materials and widely used in a variety of industrial and consumer products over past decades due to their high strength, flexibility, and durability and low manufacturing cost (Andrady and Neal, 2009). However, the accumulation of these non-degradable plastic wastes brings harmful impact on the environment for developing a sustainable society since a substantial amount of them has been disposed into ocean and landfills (Al-Salem et al., 2009). In order to address this grand challenge, tremendous efforts have been focused on the development of sustainable polymers toward an environmentally friendly future (Schneiderman and Hillmyer, 2017). Among various materials that have been proposed as promising candidates, polylactic acid (PLA) currently holds the leading position with the largest industrial production scale and explosive market growth (Murariu and Dubois, 2016), which has received significant attention for various applications such as biodegradable thermosets (Li et al., 2019), drug delivery (Casalini et al., 2019), shape memory materials (Cai et al., 2019), and food packaging (Bai et al., 2014; Tawakkal et al., 2014). However, key bottlenecks still remain for fully utilizing its potential for implementation into wide practical applications, which are associated with their inherently unsatisfactory material performances including low heating distortion temperature, brittleness, and poor thermal and hydrolytic stability (Rasal et al., 2010; Ren, 2010). The most common and robust solution to produce reinforced PLA materials is *via* stereocomplex crystallization using enantiomeric pairs of poly (L-lactide) (PLLA) and poly (D-lactide) (PDLA), which can occur from both melt and solution state (Ikada et al., 1987). For a well-mixed PLLA and PDLA blend, multicenter hydrogen bonding interactions lead to a possible alternative arrangement of helical chains between L-lactyl and D-lactyl units with opposite chiral conformation for PLA stereocomplexation (SC) (Okihara et al., 2006; Wan et al., 2019). As opposed to homocrystallites (HCs) from enantiopure PLLA or PDLA, the blend of enantiomeric PLLA and PDLA provides enhanced intermolecular interactions through dipole–dipole interaction and hydrogen bonding, leading to more tightly packed chain conformations in a side-by-side manner within the SC crystal lattice (Tsuji, 2005), thus improving mechanical property, thermal stability, and hydrolytic resistance (Tsuji and Ikada, 1999). PLA-SC exhibits a melting temperature of ~230°C, which is at least 50°C higher than the HC from neat PLLA or PDLA (Tsuji and Fukui, 2003). The hydrolytic degradation of PLA-SC is more retarded (more than three times slower) than PLA-HC when immersed in a water and phosphate buffer solution (Tsuji, 2000; Karst and Yang, 2006). Tensile strength, Young's modulus, and elongation-at-break of PLA-SC film all exhibit almost two times higher than those of homopolymer film since 3D micro crystal networks from SCs serve as intermolecular crosslinks for mechanical property enhancement (Shirahama et al., 2005). These results all indicate the significance of forming SC within PLA matrix for material property improvement and in general, a higher degree of crystallinity can provide better material physicochemical properties. Such improvement of chemical and thermal stability enables several practical applications of PLA-based materials in different fields including medicine, adhesives, and agricultural materials. As an example, the dimensional stability of PLA-SC bioplastics to heat (80°C for 30 s under 200 g load) increases nearly 4-fold (from 22 to 80%) compared with PLA-HC films (Lim et al., 2008). Water vapor permeability of PLA-SC film is also found to be 14–23% lower than that of homopolymer film (pure PLLA or PDLA), suggesting its potential applications in coating and packaging materials (Hortos et al., 2019).

**Figure 1:**
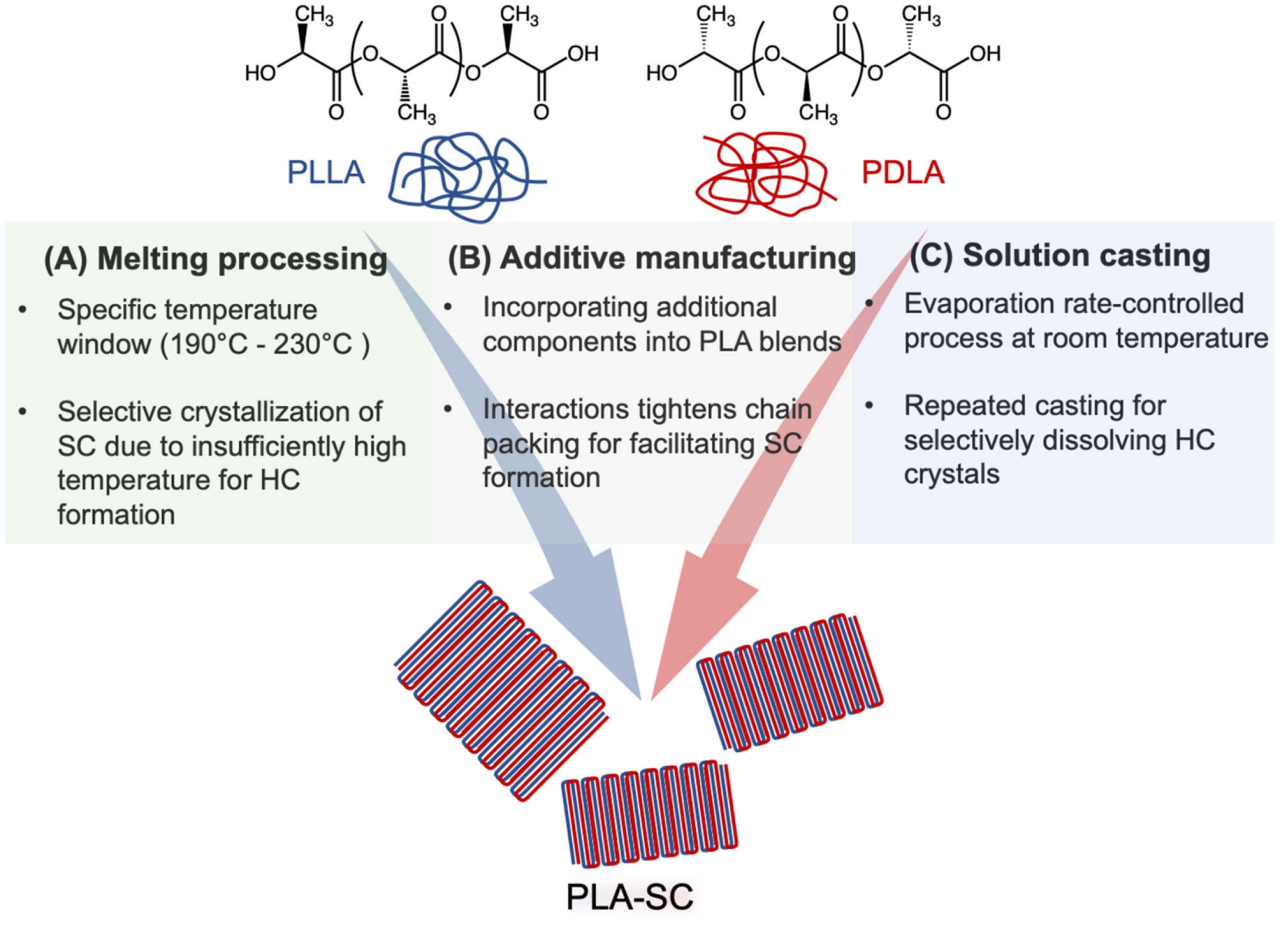
Schematic illustration of different processing methods that have been developed for enhancing the stereocomplex (SC) formation from PLLA and PDLA, which are primarily composed of either one or multiple of following strategies: **(A)** melt processing; **(B)** additive manufacturing, and **(C)** solution casting.

However, achieving the ideal scenario of exclusively forming highly crystalline SC in PLA is not a straightforward pathway. Difficulties date back to the first report of PLA-SC formation by Ikada et al. in the 1980s (Ikada et al., 1987). Conventional methods for polymer processing such as melt blowing and injection molding require a high processing temperature (220–260°C) and often yield a relatively low SC content for commercial high molecular weight (MW) PLA (Katti and Schultz, 1982; Tsuji and Tezuka, 2004; Tsuji, 2016). The challenge arises from the relatively poor thermal stability of PLA, leading to potential material decomposition when the temperature is above 230°C and the competition between simultaneous formation of SC and HC. From a kinetic perspective, HC is usually more dominant during PLA crystallization due to its lower energy barrier and has a less prolonged and restricted chain diffusion than forming SC. While a wide variety of approaches have been demonstrated to enhance the SC formation within PLLA/PDLA blend (Bai H. et al., 2017), advanced processing techniques that can offer distinct advantages of scalability and low cost for raw materials are more relevant for industrial applications compared with molecular-level engineering of PLA that involves sophisticated chemistry for controlling MW and chain architecture. Herein, this review focuses on discussing recent progress in the development of methodologies for preparing PLA-SC (Figure 1), which not only includes scientific mechanisms for the origin of PLA-SC formation enhancement from each method but also highlights their processing-structure-property relationship for different applications including engineering plastics, biomedicine, and bioelectronics. We want to note, as a mini-review, there is no attempt to be comprehensive, and interested readers can find many excellent reviews on relevant topics of PLA-based materials including material synthesis, processing, macroscopic property, and applications that provide a more in-depth discussion (Pan et al., 2017, 2018; Li et al., 2018). Our goal is rather to provide a succinct summary about progress to date on technology development for enhancing PLA-SC formation in order to compel new or interdisciplinary researchers to enter the fray and inspire more progress in the field. Therefore, we also briefly discuss the challenges that need to be addressed in this field in order to allow further advancement of PLA-based materials that warrants a green and sustainable future.

## Melt Processing at Specific Temperature Window

**Figure 2:**
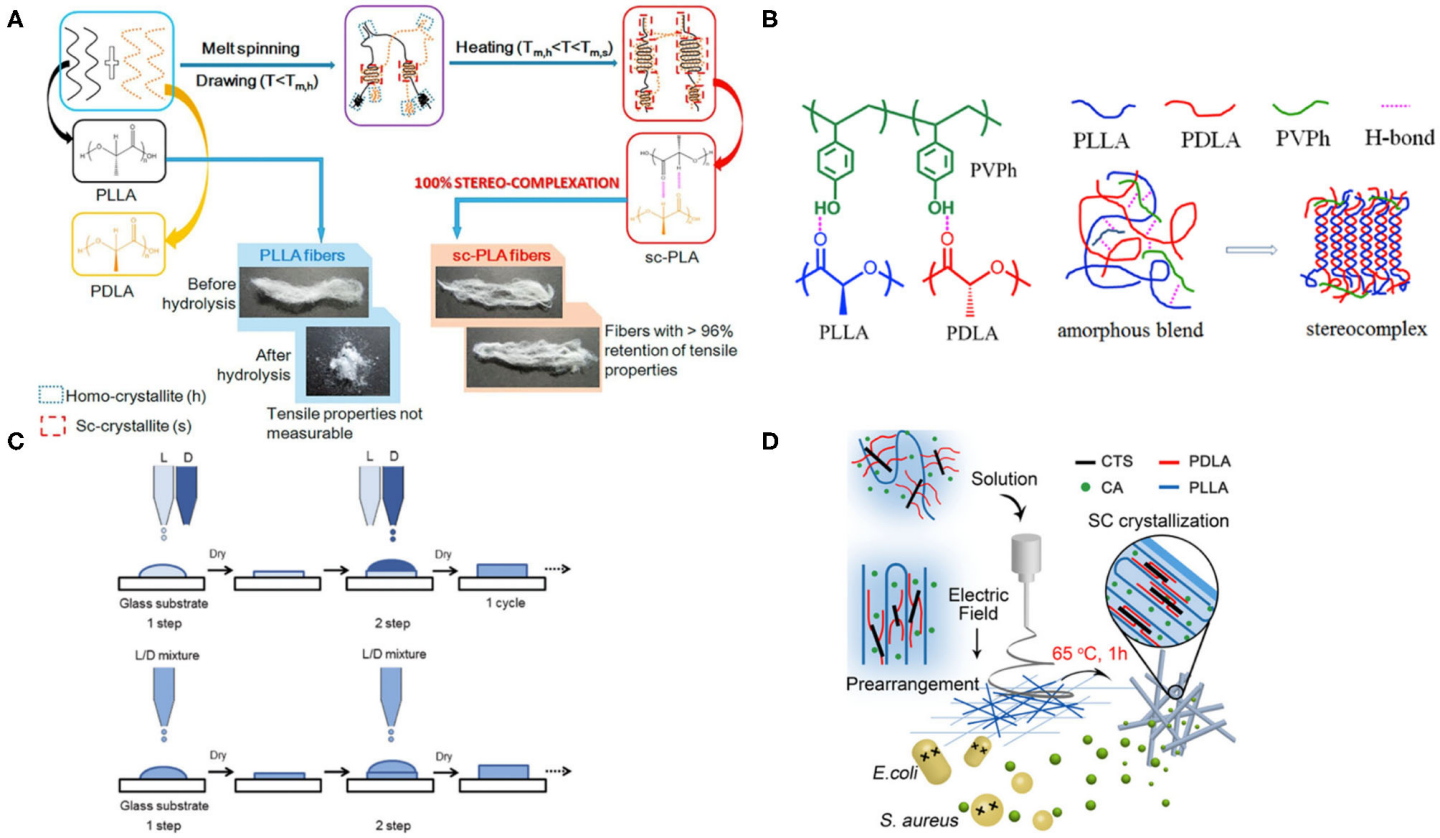
Schematic illustration of some representative processing techniques for preparing PLA materials with high degree of SC formation. **(A)** melt processing at a specific temperature window (Gao et al., 2019) (Reproduced with permission. Copyright 2017, Elsevier); **(B)** using homopolymer (He et al., 2019) (Reproduced with permission. Copyright 2016, Elsevier); **(C)** layer-by-layer inkjet printing (Mei et al., 2018) (Reproduced with permission. Copyright 2012, Wiley); and **(D)** electrospinning (Yu et al., 2018) (Reproduced with permission. Copyright 2018, ACS).

Temperature is one of the most important processing parameters to control the crystallization behavior of semicrystalline polymers (Zhang et al., 2017). PLA-SC formation can occur from either melt state by melt crystallization or glassy state by cold crystallization from PLLA/PDLA blend (Cui et al., 2018). During PLA crystallization, a higher temperature may favor to produce SCs and suppress the formation of HCs, and therefore the difference in melting temperature between SC and HC, enabling a unique processing window (190–230°C) for predominant SC formation in PLA blend. Thermal annealing of PLLA/PDLA blend at this temperature range can also induce HC to SC transition for obtaining complete SCs (Figure 2A) because HC in the PLA matrix is melted at the specific temperature while SC can still be retained, which renders partial PLA melt with improved chain mobilty to form new SCs *via* molecular rearrangements (Bai et al., 2018; Gao et al., 2019). During this process, the content of SC gradually increases with extending annealing time until a complete SC formation achieved in the matrix. This method is also effective when a mismatch in molecular weight of PLLA and PDLA exists for stereocomplexation. Complete SC formation can be achieved from a high MW PLLA/low MW PDLA blend by thermal annealing, leading to improved hydrolytic resistance and mechanical properties from enhanced molecular packing between polymer chains in the exclusive presence of SC (Liu et al., 2019). Imposing external forces on PLLA/PDLA samples such as using oscillation shear injection molding (OSIM) for intense shear also induces higher crystallinity of SCs in comparison with conventional injection molding (Bai D. et al., 2016; Bai D.-Y. et al., 2016). The kinetics of SC formation can be improved by decreasing the viscoity of polymer melt during processing such as inclusion of a low T_g_, biodegradable polymer, for reducing the activation energy of PLA melt flow for shear-thinning in order to facilitate the exclusive SC formation with high degree of crystallinity (Fu et al., 2018). Furthermore, a low-temperature sintering method for high MW PLLA/PDLA blend, involving densification, welding, and co-crystallization of powder particles, is developed to improve the degree of SC formation at a relatively low melt processing temperature of ~180°C (Bai D. et al., 2017; Ji et al., 2019). This compounding technology, inspired by powder metallurgy to produce near net metallic particles, enables improved interdiffusion of PLA chains between adjacent particles in order to co-crystallize into SCs for tightly welding the interfaces (Pan et al., 2016; Zhang et al., 2018). The formed crystalline network, which is primarily located at particle-particle interfaces, imparts the sinteredPLA-SC with enhanced mechanical strength and hydrolytic stability, excellent heat resistance, and high optical transparency. Fu et al. further broaden the application of low-temperature sintering by injection molding of partially melted nascent SC-PLA powder with small amounts of glyceryl monostearate incorporated as a lubricant (Ma et al., 2015a; Zhang et al., 2020). The non-melted SC crystallites act as nucleating agents to induce exclusive SC crystallization across the interface of the particles and accelerated the crystallization kinetics. Utilizing this method, a significantly reduced sintering time of 2 min and the ability to fabricate complex shapes like gears are achieved.

## Additive Manufacturing

Many types of additives such as compatibilizers and nucleating agents have been utilized for improving the degree of PLA-SC formation *via* providing heterogeneous nucleation surfaces and promoting chain diffusion kinetics (Wu et al., 2019). Introducing physical interactions between PLA and additives is also effective for enhancing the intermolecular packing of PLLA and PDLA chains. For example, in a miscible ternary polymer blend (PLLA/PDLA/polyvinyl phenol, or PVPh, Figure 2B), an exclusive SC formation can be achieved by employing ~30 wt% of PVPh in the system (He et al., 2019). This significant improvement is attributed to the enhanced intermolecular interactions at the initial stage of SC crystallization due to hydrogen bonding interactions between PVPh and PLLA, which not only act as precursors for facilitating SC formation but also shorten the diffusion pathway of enantiomeric chains for crystal growth. Zhang et al. demonstrate a pressure-controlled process for manipulating the morphology of SC crystals within PLLA/PDLA/PDLLA ternary blend in order to achieve combinatorial properties of improved heat resistance and faster hydrolytic degradation, providing a promising solution to on-demand tailorPLA-SC properties for a specific application (Liu et al., 2020). Confined SC formation within a cellulose template also leads to improved distortion temperature of PLA blends at 5% strain from 90 to 221°C and high specific moduli of 288.9. MPa/g from enhanced stereocomplexation due to synergesitc effects of promoted racemic helical pair formation and facilitated SC nucleation, which are both induced from the interfacial hydrogen bonding interations between cellulose and PLA chains (Brzezinski and Biela, 2014). The SC formation is further improved when polymer chains are chemically grafted onto nanoparticles or other components. In a PLLA-grafted nanocrystal cellulose (PLLA-NCC) /PDLA composite, a much higher degree of SC crystallinity (~0.5), is obtained compared with PLLA/NCC/PDLA physical blend (~0.06) after a rapid cooling from 250°C at 50°C/min. A leading explanation for this phenomenon is that PDLA molecules could assemble more easily with the bonded PLLA chains, resulting in SC with preferred closely packed geometry (Wang et al., 2020). Supertough and highly heat-resistant PLA composites are prepared by stereocomplexation between PLLA and ethylene–vinyl acetate–glycidyl methacrylate grafted PDLA (PLLA/EVMG-g-PDLA), in which EVMG elastomer is used to improve interfacial adhesion, control melt matrix viscosity for obtaining co-continuous morphology, as well as facilitate SC formation (Tsuji et al., 1991). Similarly, melt blending of the equimolar PLLA/PDLA with reactive poly (ethylene–methyl acrylate–glycidyl methacrylate) (E-MA-GMA) results in a significant increase in softening temperature (from 151 to 201°C) and heat deflection temperature (from 121 to 174°C), which can be attributed to the exclusive SC crystallite formation from *in-situ* grafting of PLA chains onto epoxy units from E-MA-GMA backbone (Tsuji and Yamamoto, 2011). Additive manufacturing stratgey can be combined with low-temperature sintering method to further enhance the formation of SC crystallites at a relatively low melt processing temperature. He et al. introduced a trace amount of untwisted carbon nanotubes (CNTs) onto PLA-SC particle surfaces, and these unentangled CNTs act as nucleating agents for promoting SC formation of PLLA/PDLA homopolymer blend on the surfaces, as well as favor SC crystals to strongly weld the interfaces during low pressure sintering process (Akagi et al., 2012). As a result, the tensile strength of PLA-SC film significantly increases from 45.8 MPa (no CNT additive) to 67.2 MPa with using only 0.001 wt% CNT additives and a linear relationship was reported between its mechanical strength and SC crystallite content. The inclusion of nanoparticles into PLA-SC matrix also enables additional functionality for more diverse applications (Becker et al., 2010). For example, an optimized electrical conductivity of PLA-SC composites can be achieved from the introduction of multiwall carbon nanotubes (MWCNTs) with a continuous domain morphology to facilitate electron transportation (Hang Thi et al., 2017). Alternatively, embedding silver nanowires (AgNW) into PLLA/PDLA blend significantly improves the electric conductivity for optoelectronic devices that are stable over 10,000 bending cycles while enhancing tensile strength, modulus, and heat resistance from SC formation. The organic light-emitting diode (OLED) prepared from the optimal AgNW/PLA-SC composite exhibits high flexibility and luminosity, suggesting the great potential of PLA-SC based materials in bioelectronic applications (Koide et al., 2017).

## Solution Casting

Since melt processing of PLA imposes potential concerns about thermal degradation and property deterioration, solution-casting is often used as an alternative to prepare PLA-SC from PLLA/PDLA homopolymer blend solution, followed by solvent evaporation at room temperature. During this dynamic process, an exclusive SC formation can occur at a specific concentration range between critical solution concentrations for SC and HC formation (c_SC_^*^ < c < c_HC_*) (Gardella et al., 2015). A sufficiently slow solvent evaporation rate likely yields a high content of SC in the blend, but it is not desired for industrial production rate/volume due to its inevitably long processing time. To address this challenge, a method involving repeated castings of PLLA/PDLA blend is developed to enhance the degree of SC crystallization by utilizing the solubility difference between HC and SC in the solvent so that only HCs are dissolved during sequential casting while preserving previously formed SCs (Ma et al., 2015b). A multiple casting strategy significantly increases the content of SCs and eventually leads to almost no presence of HC in the films. The concept of simultaneous casting and selective dissolution of HCs can be extended to a layer-by-layer casting approach (Figure 2C) through alternative deposition of PLLA and PDLA homopolymers onto the substrate using inkjet printing technique for achieving a predominant SC formation (Mei et al., 2018; Xie et al., 2018). Hang et al. deomonstrate that the thermal decomposition temperature of PLA-SC formed *via* this inkjet printing approach shows an increase of more than 90°C compared with original PLA without inclusion of SCs (Im et al., 2018). The layer-by-layer deposition process is suitable for manufacturing three-dimensional polymer composites with a customizable material shape. Strategies including adding small amounts of non-solvent and using ionic liquids also facilitate the formation of SCs in the polymer blends (Ishii et al., 2009; Jing et al., 2016). Furthermore, an additional thermal annealing step for cold crystallization of these as-cast films would be useful for further improving their thermal stability and mechanical properties from the nucleating effect of SC crystallines in PLA matrix (Boi et al., 2019b). The enantiomeric polymer blend solution can be used for other processing techniques such as electrospining and templated-drying in order to produce SC crystal embedded PLA with complex macroscopic shapes and enhanced final material properties. Electrospinning of PLA blend solution is able to produce a high degree of SC content due to the improved intermolecular ordering between PLLA and PDLA chains from high shear force induced by electric field (Figure 2D) (Yu et al., 2018). Strengthened PLA-based materials with improved thermal (melting temprature over 200°C) and mechanical properteis (Young's modulus up to 270 MPa) are prepared by the electrospinning of a PDLA-grafted chitosan (PDLA-CTC) /PLLA mixture, in which SC formation is assisted by intermolecular hydrogen bonding interactions between stereoisomers. Additionally, Xie et al. demonstrate a simple method for fabricating biodegradable PLA-SC porous scaffolds by solution casting of salt-embedding PLLA/PDLA blend, in which the salt particles (sodium chloride) are served as pore-forming agents by leaching. The obtained foam shows an improved thermal stability with a high structural intergrity even at elevated temperatures (~120°C) due to the presence of PLA-SCs (Boi et al., 2019a). It is also very important to point out that these solution-based processing methods lead to additional environmental and safety concerns due to the requirement of using large amounts of volatile and toxic organics solvents, so more environmentally friendly solvents such as supercritical fluid should be considered in the future (Cui et al., 2013).

## Property Improvement for Biomedical Applications

Besides being an alternative for conventional plastics, PLA-SC holds great promise for use in medical applications such as tissue scaffold and membrane for cell growth or proliferation due to its improved biocompatibility and bioresorbability compared with HC from analogous PLLA and PDLA (Tobió et al., 1998). An *in vivo* study from Ishii et al. demonstrates a slower degradation rate and a much milder inflammation of PLA-SC fibers than PLLA homopolymer fibers after 12 weeks of subcutaneous implantation in rats (Campana et al., 2014). PLA-SC can also serve as universal carriers for drugs (Ouchi and Ohya, 2004), proteins, and vaccines with the programmable release kinetics controlled by its microstructure (Oh, 2011; Dong et al., 2020). Furthermore, micellar particles self-assembled from stereocomplexation of PLA-based block copolymers have been commonly used in biomaterial research for injectable hydrogel (Nguyen and Lee, 2010), drug delivery (Feng et al., 2016), advanced diagnosis and treatment (Kersey et al., 2010), imaging agents, and antibacterial coating (Spasova et al., 2010; Qi et al., 2019).

## Conclusions and Perspectives

In conclusion, the stereocomplexation (SC) of PLLA and PDLA homopolymers is a very effective strategy for enhancing the properties of PLA-based materials in order to overcome their inherent limitations, which include poor thermal and hydrolytic stability, brittleness, and insufficient toughness. This review highlights recent advances in material preparation and processing technologies for enhancing SC crystallization, which is a key parameter for material property improvement. It is notable that even with these remarkable developments, there are still some challenges that remain unresolved for enabling wide applications of PLA-SC in biomedicine, commodity, and engineering plastics. For example, (1) the high cost of PDLA has been a major obstacle for large-scale industrial manufacturing, so the development of high-performance PLA-SC with a low PDLA content is beneficial for reducing production costs; (2) research has been intensively focused on controlling the structure and property of PLA-SC blends, but studies about quantitatively understanding their biodegradability and associated degradation kinetics upon local environment are still limited. A whole life cycle analysis of PLA products would be useful for promoting the development of relevant industrial chains; (3) the development of engineering solutions for on-demand manipulating crystal morphology of PLA-SCs would be essential for controlling ultimate material performance; (4) finally, exciting new opportunities have recently emerged for degradable bioelectronic applications for health monitoring and advanced therapeutic technologies (Tan et al., 2016), by which PLA has much to offer in this context. The continuous research progress in these areas is anticipated for enabling a more significant role of PLA-based materials toward a sustainable development of life and technology in the future.

## Author Contributions

All authors listed have made a substantial, direct and intellectual contribution to the work, and approved it for publication.

## Conflict of Interest

The authors declare that the research was conducted in the absence of any commercial or financial relationships that could be construed as a potential conflict of interest.
